# Conceptual DFT Study of the Local Chemical Reactivity of the Colored BISARG Melanoidin and Its Protonated Derivative

**DOI:** 10.3389/fchem.2018.00136

**Published:** 2018-05-01

**Authors:** Juan Frau, Daniel Glossman-Mitnik

**Affiliations:** ^1^Departament de Química, Universitat de les Illes Balears Palma de Mallorca, Spain; ^2^Laboratorio Virtual NANOCOSMOS, Centro de Investigación en Materiales Avanzados, Departamento de Medio Ambiente y Energía, Chihuahua, Mexico

**Keywords:** BISARG, conceptual DFT, chemical reactivity, dual descriptor, Parr functions

## Abstract

This computational study assessed eight fixed RSH (range-separated hybrid) density functionals that include CAM-B3LYP, LC-ωPBE, M11, MN12SX, N12SX, ωB97, ωB97X, and ωB97XD related to the Def2TZVP basis sets together with the SMD solvation model in the calculation the molecular structure and reactivity properties of the BISARG intermediate melanoidin pigment (5-(2-(E)-(Z)-5-[(2-furyl)methylidene]-3-(4-acetylamino-4-carboxybutyl)-2-imino-1,3-dihydroimidazol-4-ylideneamino(E)-4-[(2-furyl)methylidene]-5-oxo-1H-imidazol-1-yl)-2-acetylaminovaleric acid) and its protonated derivative, BISARG(p). The chemical reactivity descriptors for the systems were calculated via the Conceptual Density Functional Theory. The choice of active sites applicable to nucleophilic, electrophilic as well as radical attacks were made by linking them with Fukui functions indices, electrophilic and nucleophilic Parr functions, and the condensed Dual Descriptor Δf(**r**). The study found the MN12SX and N12SX density functionals to be the most appropriate in predicting the chemical reactivity of the molecular systems under study starting from the knowledge of the HOMO, LUMO, and HOMO-LUMO gap energies.

## 1. Introduction

Visual color in processed foods is largely due to colored products of Maillard or nonenzymic browning reactions. In spite of the longstanding aesthetic and practical interest in Maillard derived food coloring materials, relatively little is known about the chemical structures responsible for visual color (Rizzi, 1997). These chemical structures are known as Colored Maillard Reaction Products and can be isolated at intermediate stages during the melanoidin formation process.

Besides their interest as dye molecules which may be useful as food additives, but also as dyes for dye-sensitized solar cells (DSSC), these compounds have also antioxidant capabilities. Thus, they are amenable to be studied by analyzing their molecular reactivity properties.

One of these isolated molecules is named by the acronym BISARG and together with its protonated derivative, BISARG(p) have been experimentally studied as a part of a work related to the formation of melanidins (Hofmann, 1998) and we believe that it could be of interest to study their molecular reactivity by using the ideas of Conceptual DFT, in the same way of our previous works (Alvarado-González et al., 2013; Cervantes-Navarro and Glossman-Mitnik, 2013; Glossman-Mitnik, 2013a,b; Martínez-Araya et al., 2013a,b; Salgado-Morán et al., 2013; Glossman-Mitnik, 2014a,b,c,d; Martínez-Araya and Glossman-Mitnik, 2015; Martínez-Araya et al., 2015; Soto-Rojo et al., 2015; Frau et al., 2016a,b,c; Mendoza-Huízar et al., 2016; Frau et al., 2017a,b,c,d,e; Frau and Glossman-Mitnik, 2017a,b,c,d,e,f,g; Sastre et al., 2017).

The interest in using range-separated (RS) exchange correlation functionals in KS DFT is on the rise (Gledhill et al., 2016). The functionals tend to partition the ${\text{r}}_{12}^{-1}$ operator and exchange them into long- and short-range parts, whose range separation parameter ω controls the rate of attaining the long-range behavior. It is possible to fix the value of ω. The value can also be nonempirically “tuned” through a system-by-system mechanism that minimizes some tuning norms. The basis of the optimal tuning approach is the knowledge that the energy that the HOMO should have, ϵ_*H*_(N), in exact KS as well as generalized KS theory for an N electron system, ought to be exactly −IP(N). Hence, IP represents the vertical ionization potential that is calculated by considering a particular functional energy difference E(N-1) − E(N). If approximate functionals are used, it is possible to have considerable differences between ϵ_*H*_(N), and −IP(N). Optimal tuning constitutes determining a system-specific range-separation parameter ω non-empirically in an RSE functional. Optionally, it also implied that several other parameters including ϵ_*H*_(N) = −IP(N) are satisfied optimally (Jacquemin et al., 2014). Even though no equivalency exists to match this prescription of electron affinity (EA) coupled with LUMO in the case of neutral species, it is possible to say that ϵ_*H*_(N+1) = −EA(N), that is, the electron affinity of the neutral system is equal to minus the HOMO energy of the anion (SOMO), which facilitates the finding of an optimized value of ω, and is then optimized to establish both properties simultaneously. Some concerns have been raised during the preparation of this paper regarding the validity of the ionization potential theorem (IP) within the context of Generalized Kohn-Sham (GKS) theory. However, it must stressed that Baer et al. (2010) and more recently Baerends et al. (2013) and Karolewski et al. (2013) have given arguments that the same criterion applies in GKS theories and with with hybrid and range-separated hybrid functionals. This will make it easy to predict the Conceptual DFT descriptors. In the past, the simultaneous prescription has been referred to as the “KID procedure” (for Koopmans in DFT), courtesy of the analogy it shares with the Koopmans' theorem within the Hartree-Fock theory. This SOMO energy will not be, in general, equal to the LUMO of the neutral, but if the difference between them, which we have called ΔSL, is small enough to be considered negligible for predictions of the Conceptual DFT descriptors, then the practical KID procedure will have a computational support.

This implies that the appropriateness of a particular density functional in making predictions of the Conceptual DFT descriptors directly by relying on the properties that the neutral molecule can be easily estimated. It only requires one to check the way that it has followed the KID procedure. Nevertheless, tune-optimization depends on the system and must be performed for each molecule one at a time. Therefore, examining the various density functionals exhibiting significant accuracy across various types of databases in physics, chemistry, and where the ω value is fixed will determine how they perform the practical technique.

Thus, in this computational study we will assess eight density functionals in calculating the molecular properties and structure of the BISARG intermediate melanoidin pigment and its protonated derivative, BISARG(p). Following the same ideas of previous works, we will consider fixed RSH functional instead of the optimally-tuned RSH density functionals that have attained great success and have also supported the validity of the IP theorem in the context of the GKS theory (Stein et al., 2009a,b; Karolewski et al., 2011; Kuritz et al., 2011; Refaely-Abramson et al., 2011; Foster and Wong, 2012; Koppen et al., 2012; Kronik et al., 2012; Phillips et al., 2012a,b; Karolewski et al., 2013; Moore and Autschbach, 2013; Egger et al., 2014; Foster et al., 2014; Jacquemin et al., 2014; Niskanen and Hukka, 2014; Sun and Autschbach, 2014; Manna et al., 2015; Lima et al., 2016; Pereira et al., 2017).

## 2. Theoretical background

The theoretical background of this study is similar to the previous conducted research presented complete purposes, because this research is a component of a major project that it is in progress. If we consider the KID procedure mentioned in the Introduction together with a finite difference approximation, then the global reactivity descriptors can be written as:

**Table:** 

Electronegativity	$\chi =-\frac{1}{2}\left(I+A\right)\approx \frac{1}{2}\left(\epsilon_{L}+\epsilon_{H}\right)$	Parr and Yang (1984)

Global Hardness	η = (*I* − *A*) ≈ (ϵ_*L*_ − ϵ_*H*_)	Parr and Yang (1984)

Electrophilicty	$\omega =\frac{\mu^{2}}{2\eta }=\frac{{\left(I+A\right)}^{2}}{4\left(I-A\right)}\approx \frac{{\left(\epsilon_{L}+\epsilon_{H}\right)}^{2}}{4\left(\epsilon_{L}-\epsilon_{H}\right)}$	Parr et al. (1999)

Electrodonating Power	$\omega^{-}=\frac{{\left(3I+A\right)}^{2}}{16\left(I-A\right)}\approx \frac{{\left(3\epsilon_{H}+\epsilon_{L}\right)}^{2}}{16\eta }$	Gázquez et al. (2007)

Electroaccepting Power	$\omega^{+}=\frac{{\left(I+3A\right)}^{2}}{16\left(I-A\right)}\approx \frac{{\left(\epsilon_{H}+3\epsilon_{L}\right)}^{2}}{16\eta }$	Gázquez et al. (2007)

Net electrophilicity	*Δω*^±^ = ω^+^ − (−ω^−^) = ω^+^ + ω^−^	Chattaraj et al. (2009)

where ϵ_*H*_ and ϵ_*L*_ are the energies of the highest occupied and the lowest unoccupied molecular orbitals (HOMO and LUMO), respectively.

Applying the same ideas, the definitions for the local reactivity descriptors are:

**Table:** 

Nucleophilic Fukui Function	$f^{+}\left(\text{r}\right)=\rho_{N+1}\left(\text{r}\right)-\rho_{N}\left(\text{r}\right)$	Parr and Yang (1984)

Electrophilic Fukui Function	$f^{-}\left(\text{r}\right)=\rho_{N}\left(\text{r}\right)-\rho_{N-1}\left(\text{r}\right)$	Parr and Yang (1984)

Dual Descriptor	$\Delta f\left(\text{r}\right)={\left(\frac{\partial f\left(\text{r}\right)}{\partial N}\right)}_{\upsilon \left(\text{r}\right)}$	Morell et al. (2005, 2006)

Nucleophilic Parr Function	$P^{-}\left(\text{r}\right)=\rho_{s}^{rc}\left(\text{r}\right)$	Domingo et al. (2013)

Electrophilic Parr Function	$P^{+}\left(\text{r}\right)=\rho_{s}^{ra}\left(\text{r}\right)$	Domingo et al. (2013)

where ρ_*N*+1_(**r**), ρ_*N*_(**r**), and ρ_*N*−1_(**r**) are the electronic densities at point **r** for the system with *N* + 1, *N*, and *N* − 1 electrons, respectively, and $\rho_{s}^{rc}\left(\text{r}\right)$ and $\rho_{s}^{ra}$ (**r**) are related to the atomic spin density (ASD) at the **r** atom of the radical cation or anion of a given molecule, respectively (Domingo et al., 2016).

## 3. Settings and computational methods

Following the lines of our previous works, the computational studies were performed with the Gaussian 09 (Frisch et al., 2018) series of programs with density functional methods as implemented in the computational package. The basis set used in this work was Def2SVP for geometry optimization and frequencies, while Def2TZVP was considered for the calculation of the electronic properties (Weigend and Ahlrichs, 2005; Weigend, 2006). All the calculations were performed in the presence of water as the solvent by doing Integral Equation Formalism-Polarized Continuum Model (IEF-PCM) computations according to the SMD solvation model (Marenich et al., 2009).

For the calculation of the molecular structure and properties of the studied systems, we have chosen eight density functionals which are known to consistently provide satisfactory results for several structural and thermodynamic properties:

**Table:** 

CAM-B3LYP	Long-range-corrected B3LYP by the CAM method	Yanai et al. (2004)
LC-ωPBE	Long-range-corrected ωPBE density functional	Henderson et al. (2009)
M11	Range-separated hybrid meta-GGA	Peverati and Truhlar (2011)
MN12SX	Range-separated hybrid nonseparable meta-NGA	Peverati and Truhlar (2012)
N12SX	Range-separated hybrid NGA	Peverati and Truhlar (2012)
ωB97	Long-range corrected density functional	Chai and Head-Gordon (2008b)
ωB97X	Long-range corrected density functional	Chai and Head-Gordon (2008b)
ωB97XD	ωB97X version including empirical dispersion	Chai and Head-Gordon (2008a)

In these functionals, GGA stands for generalized gradient approximation (in which the density functional depends on the up and down spin densities and their reduced gradient) and NGA stands for nonseparable gradient approximation (in which the density functional depends on the up/down spin densities and their reduced gradient, and also adopts a nonseparable form).

## 4. Results and discussion

The three-dimentional molecular structure of the BISARG system was built with the aid of molecular graphics program starting from structure presented in the original article (Hofmann, 1998). Starting from this, the molecular structure of its protonated derivative, BISARG(p) was built with the aid of a chemical visualization software. The pre-optimization of the systems was done using random sampling that involved molecular mechanics techniques and inclusion of the various torsional angles via the general MMFF94 force field (Halgren, 1996a,b,c, 1999; Halgren and Nachbar, 1996) through the Marvin View 17.15 program that constitutes an advanced chemical viewer suited to multiple and single chemical queries, structures and reactions (https://www.chemaxon.com). Afterwards, the structures that the resultant lower-energy conformers assumed for both molecules were reoptimized using the eight density functionals mentioned in the previous section together with the Def2SVP basis set as well as the SMD solvation model using water as the solvent.

The analysis of the results obtained in the study aimed at verifying that the KID procedure was fulfilled. On doing it previously, several descriptors associated with the results that HOMO and LUMO calculations obtained are related with results obtained using the vertical I and A following the ΔSCF procedure. A link exists between the three main descriptors and the simplest conformity to the Koopmans' theorem by linking ϵ_*H*_ with -I, ϵ_*L*_ with -A, and their behavior in describing the HOMO-LUMO gap as *J*_*I*_ = |ϵ_*H*_+*E*_*gs*_(*N* − 1)−*E*_*gs*_(*N*)|, *J*_*A*_ = |ϵ_*L*_+*E*_*gs*_(*N*)−*E*_*gs*_(*N* + 1)|, and $J_{HL}=\sqrt{{J_{I}}^{2}+{J_{A}}^{2}}$. Notably, the *J*_*A*_ descriptor consists of an approximation that remains valid only when the HOMO that a radical anion has (the SOMO) shares similarity with the LUMO that the neutral system has. Consequently, we decided to design another descriptor ΔSL (the difference between the SOMO and LUMO energies), to guide in verifying how the approximation is accurate.

The results of the calculation of the electronic energies of the neutral, positive and negative molecular systems (in au) of BISARG and BISARG(p), the HOMO, LUMO, and SOMO orbital energies (in eV), *J*_*I*_, *J*_*A*_, *J*_*HL*_, and ΔSL descriptors calculated with the eight density functionals and the Def2TZVP basis set using water as solvent simulated with the SMD parametrization of the IEF-PCM model are presented in Tables 1, 2.

**Table 1:** Electronic energies of the neutral, positive and negative molecular systems (in au) of the BISARG molecule, the HOMO, LUMO, and SOMO orbital energies (in eV), *J*_*I*_, *J*_*A*_, *J*_*HL*_, and ΔSL descriptors (also in eV) calculated with the eight RSH density functionals and the Def2TZVP basis set using water as solvent simulated with the SMD parametrization of the IEF-PCM model.

	**Eo**	**E+**	**E−**	**HOMO**	**LUMO**	**SOMO**	***J*_*I*_**	***J*_*A*_**	***J*_*HL*_**	**ΔSL**
CAM-B3LYP	−2276.0764	−2275.8795	−2276.1832	−6.6124	−1.6898	−4.1310	1.2522	1.2168	1.7458	2.4412
LC-ωBPE	−2275.6818	−2275.4754	−2275.7958	−7.8736	−0.9566	−5.2792	2.2572	2.1454	3.1139	4.3226
M11	−2275.9123	−2275.7056	−2276.0238	−7.6575	−1.1204	−4.9627	2.0321	1.9146	2.7920	3.8422
MN12SX	−2275.1376	−2274.9395	−2275.2504	−5.3883	−3.0639	−3.0873	0.0022	0.0082	0.0084	0.0144
N12SX	−2276.0990	−2275.9088	−2276.2081	−5.1959	−2.9278	−3.0064	0.0218	0.0433	0.0484	0.0787
ωB97	−2276.7888	−2276.5871	−2276.8951	−7.7315	−0.7982	−5.0476	2.2420	2.0947	3.0685	4.2494
ωB97X	−2276.5890	−2276.3879	−2276.6957	−7.5543	−0.9354	−4.9069	2.0819	1.9682	2.8652	3.9715
ωB97XD	−2276.4388	−2276.2394	−2276.5463	−7.2139	−1.1972	−4.6660	1.7858	1.7271	2.4842	3.4688

**Table 2:** Electronic energies of the neutral, positive and negative molecular systems (in au) of the protonated BISARG(p) molecule, the HOMO, LUMO, and SOMO orbital energies (in eV), *J*_*I*_, *J*_*A*_, *J*_*HL*_, and ΔSL descriptors (also in eV) calculated with the eight RSH density functionals and the Def2TZVP basis set using water as solvent simulated with the SMD parametrization of the IEF-PCM model.

	**Eo**	**E+**	**E−**	**HOMO**	**LUMO**	**SOMO**	***J*_*I*_**	***J*_*A*_**	***J*_*HL*_**	**ΔSL**
CAM-B3LYP	−2276.5424	−2276.3458	−2276.6659	−6.6364	−2.0844	−4.6328	1.2862	1.2775	1.8128	2.5484
LC-ωBPE	−2276.1485	−2275.9438	−2276.2828	−7.8698	−1.3822	−5.8926	2.2969	2.2703	3.2296	4.5103
M11	−2276.3780	−2276.1724	−2276.5077	−7.6670	−1.5308	−5.5304	2.0719	2.0011	2.8804	3.9995
MN12SX	−2275.6003	−2275.4000	−2275.7267	−5.4495	−3.4299	−3.4481	0.0014	0.0114	0.0114	0.0182
N12SX	−2276.5694	−2276.3768	−2276.6925	−5.2653	−3.2944	−3.3910	0.0237	0.0558	0.0604	0.0966
ωB97	−2277.2550	−2277.0549	−2277.3813	−7.7220	−1.2241	−5.6655	2.2784	2.2150	3.1776	4.4314
ωB97X	−2277.0557	−2276.8559	−2277.1814	−7.5546	−1.3488	−5.4980	2.1179	2.0727	2.9634	4.1492
ωB97XD	−2276.9093	−2276.7104	−2277.0339	−7.2329	−1.5926	−5.1894	1.8210	1.7976	2.5588	3.5967

As presented in previous works, we considered four other descriptors that analyze how well the studied density functionals are useful for the prediction of the electronegativity χ, the global hardness η, and the global electrophilicity ω, and for a combination of these Conceptual DFT descriptors, considering only the energies of the HOMO and LUMO or the vertical I and A: *J*_χ_ = |χ − χ_*K*_|, *J*_η_ = |η − η_*K*_|, *J*_ω_ = |ω − ω_*K*_|, and $J_{CDFT}=\sqrt{J_{\chi }^{2}+J_{\eta }^{2}+J_{\omega }^{2}}$, where CDFT stands for Conceptual DFT. The underscript K stands for the descriptor calculated by applying the KID procedure.The results of the calculations of *J*_χ_, *J*_η_, *J*_ω_, and *J*_*CDFT*_ for the low-energy conformers of BISARG and BISARG(p) in water are displayed in Tables 3, 4, respectively.

**Table 3:** *J*_χ_, *J*_η_, *J*_ω_, and *J*_*CDFT*_ (in eV) of the BISARG intermediate melanoidin pigment.

	***J*_χ_**	***J*_η_**	***J*_ω_**	***J*_*CDFT*_**
CAM-B3LYP	0.0185	2.4706	1.7322	3.0174
LC-ωBPE	0.0552	4.4023	2.3704	5.0002
M11	0.0591	3.9462	2.1447	4.4917
MN12SX	0.0045	0.0030	0.0131	0.0142
N12SX	0.0109	0.0610	0.1205	0.1355
ωB97	0.0738	4.3370	2.0710	4.8067
ωB97X	0.0565	4.0499	2.0530	4.5409
ωB97XD	0.0294	3.5156	2.0168	4.0531

**Table 4:** *J*_χ_, *J*_η_, *J*_ω_, and *J*_*CDFT*_ (in eV) of the protonated BISARG(p) intermediate melanoidin pigment.

	***J*_χ_**	***J*_η_**	***J*_ω_**	***J*_*CDFT*_**
CAM-B3LYP	0.0046	2.5626	2.6800	3.7080
LC-ωBPE	0.0131	4.5716	3.9039	6.0117
M11	0.0363	4.0705	3.3156	5.2501
MN12SX	0.0059	0.0084	0.0334	0.0349
N12SX	0.0161	0.0795	0.2316	0.2454
ωB97	0.0316	4.4894	3.3713	5.6144
ωB97X	0.0225	4.1892	3.2674	5.3128
ωB97XD	0.0107	3.6182	3.0654	4.7422

As Tables 1–4 provide, the KID procedure applies accurately from MN12SX and N12SX density functionals that are range-separated hybrid meta-NGA as well as range-separated hybrid NGA density functionals respectively. In fact, the values of *J*_*I*_, *J*_*A*_, and *J*_*HL*_ are actually not zero. Nevertheless, the results tend to be impressive especially for the MN12SX density functional. As well, the ΔSL descriptor reaches the minimum values when MN12SX and N12SX density functionals are used in the calculations. This implies that there are sufficient justifications to assume that the LUMO of the neutral approximates the electron affinity. The same density functionals follow the KID procedure in the rest of the descriptors such as *J*_χ_, *J*_η_, *J*_ω_, and *J*_*CDFT*_.

Having verified that the MN12SX/Def2TZVP model chemistry is a good choice for the calculation of the global reactivity descriptors, we now present the optimized molecular structures of BISARG and BISARG(p) in water in Supplementary Figures 1, 2. Meanwhile, the calculated bond lengths and bond angles for both cases are shown in Supplementary Tables 1–4.

As a summary of the previous results, the global reactivity descriptors for the BISARG and BISARG(p) molecules calculated with the MN12SX/Def2TZVP model chemistry in water are presented in Table 5.

**Table 5:** Global reactivity descriptors for the BISARG intermediate melanoidin pigment and its protonated derivative BISARGd(p) calculated with the MN12SX density functional.

	**Electronegativity (χ)**	**Chemical hardness (η)**	**Electrophilicity (ω)**
BISARG	4.2253	2.3240	3.8410
BISARG(p)	4.4389	2.0193	4.8790
	**Electrodonating Power (**ω^−^**)**	**Electroaccepting Power (**ω^+^**)**	**Net electrophilicity (**Δω^±^**)**
BISARG	5.8449	4.4318	10.2767
BISARG(p)	7.1172	5.6637	12.7809

The calculations of the condensed Fukui functions and dual descriptor are done by using the Chemcraft molecular analysis program to extract the Mulliken and NPA atomic charges (Zhurko and Zhurko, 2012) beginning with single-point energy calculations involving the MN12SX density functional that uses the Def2TZVP basis set in line with the SMD solvation model, and water utilized as the solvent.

**Figure 1:**
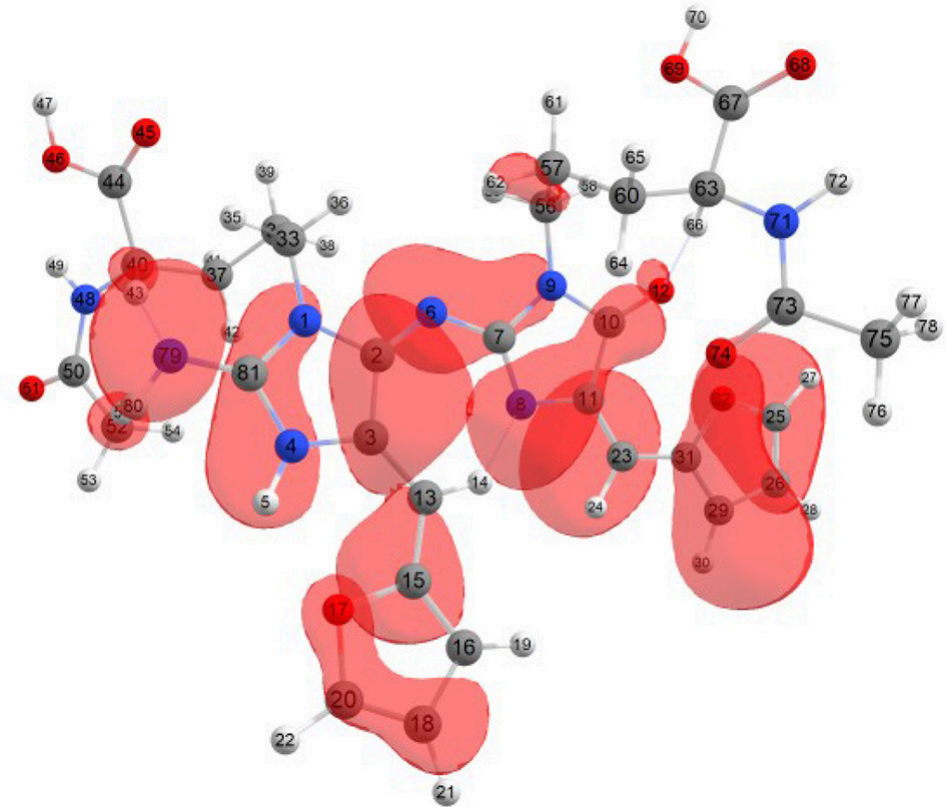
A graphical schematic representation of the radical Fukui function *f*^0^ over the atomic sites of the BISARG intermediate melanoidin pigment.

**Figure 2:**
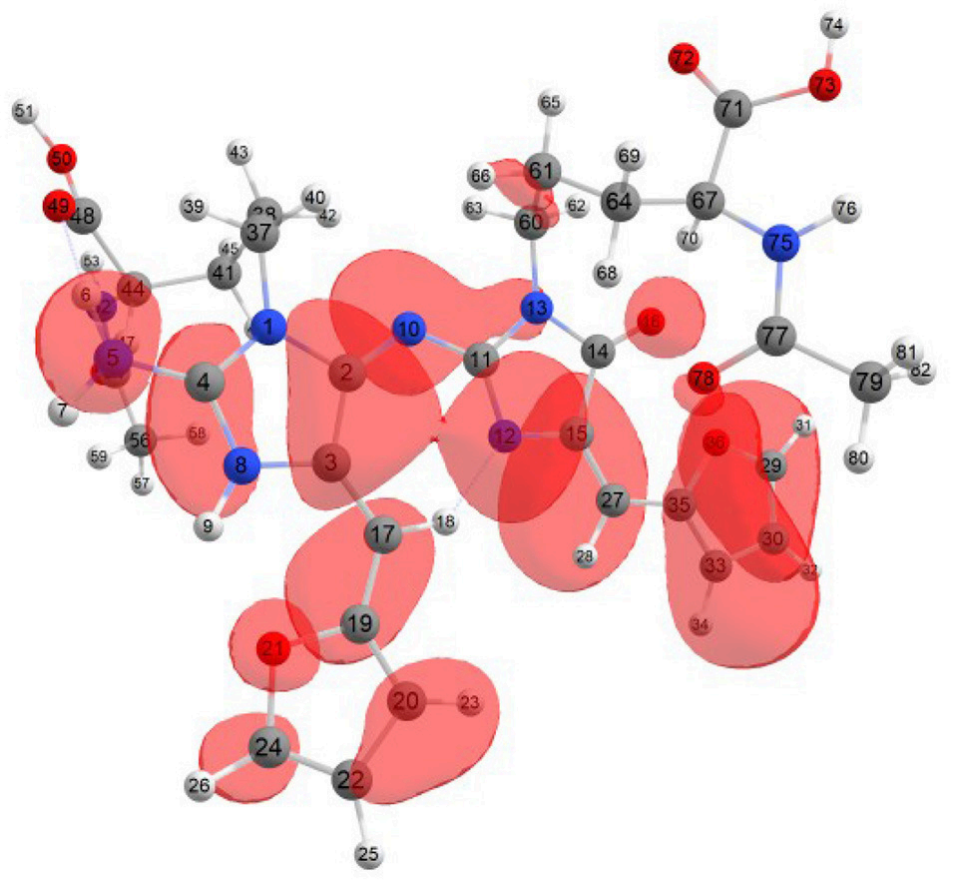
A graphical schematic representation of the radical Fukui function *f*^0^ over the atomic sites of the protonated BISARG(p) intermediate melanoidin pigment.

Considering the potential application the studied molecules as antioxidants, it is of interest to get insight into the active sites for radical attack. Graphical representations of the radical Fukui function *f*^0^ calculated with the MN12SX/Def2TZVP model chemistry for both systems in water are presented in Figures 1, 2.

The condensed electrophilic and nucleophilic Parr functions $P_{k}^{+}$ and $P_{k}^{-}$ over the atoms of the BISARG and BISARG(p) molecules in water have been calculated by extracting the Mulliken and Hirshfeld (or CM5) atomic charges using the Chemcraft molecular analysis program (Zhurko and Zhurko, 2012) starting from single-point energy calculations of the ionic species with the MN12SX density functional using the Def2TZVP basis set in the presence of the solvents according to the SMD solvation model.

**Figure 3:**
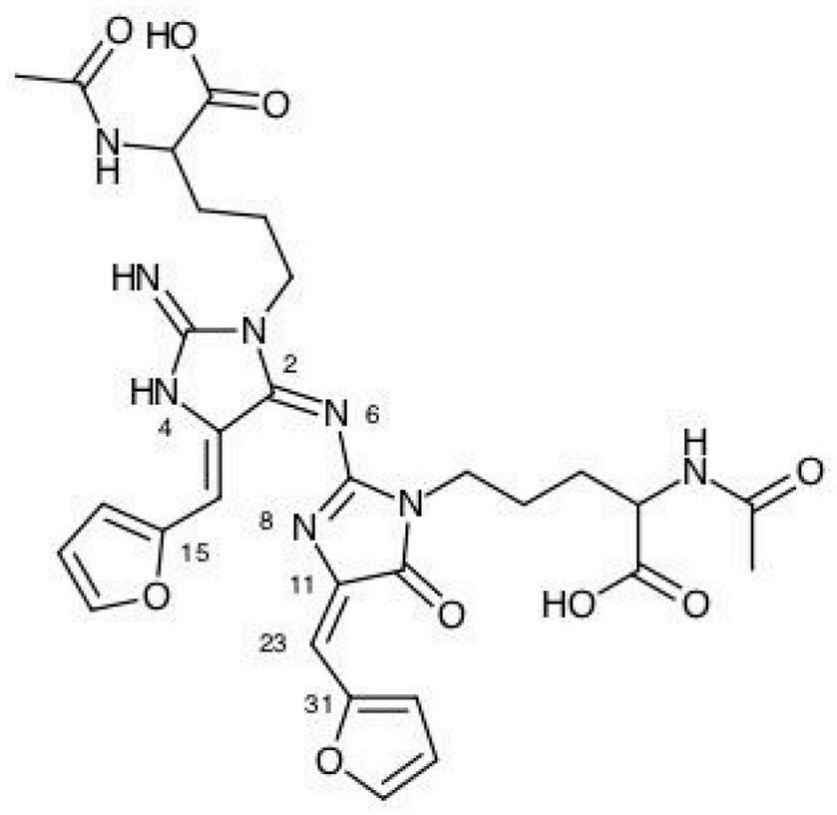
A graphical schematic representation of the BISARG intermediate melanoidin pigment with the numbering of the most important reactive sites.

**Figure 4:**
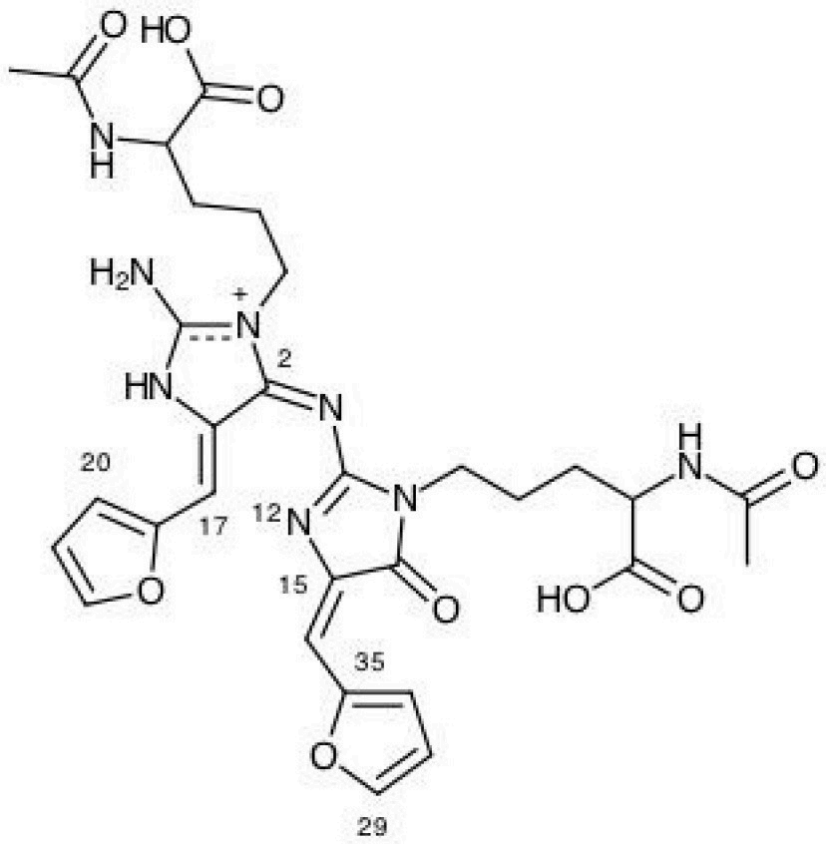
A graphical schematic representation of the protonated BISARG(d) intermediate melanoidin pigment with the numbering of the most important reactive sites.

The results for the condensed dual descriptor calculated with Mulliken atomic charges Δf_*k*_ (M), with NPA atomic charges Δf_*k*_ (N), the electrophilic and nucleophilic Parr functions with Mulliken atomic spin densities $P_{k}^{+}$ (M) and $P_{k}^{-}$ (M), and the electrophilic and nucleophilic Parr functions with Hirshfeld (or CM5) atomic spin densities $P_{k}^{+}$ (H) and $P_{k}^{-}$ (H) are displayed in Tables 6, 7 for the BISARG and BISARG(p) molecules in water, respectively, while Figures 3, 4 show schematic representations of the molecules with the numbering of the most important reactive sites according to the results in Tables 6, 7.

**Table 6:** The condensed dual descriptor calculated with Mulliken atomic charges Δf_*k*_ (M), and with NPA atomic charges Δf_*k*_ (N), the electrophilic and nucleophilic Parr functions with Mulliken atomic spin densities $P_{k}^{+}$ (M) and $P_{k}^{-}$ (M), and the electrophilic and nucleophilic Parr functions with Hirshfeld (or CM5) atomic spin densities $P_{k}^{+}$ (H) and $P_{k}^{-}$ (H) for the BISARG melanoidin molecule. Hydrogens and atomic sites where the absolute value of the dual descriptor is lower than 1 are not shown.

**Atom**	**Δf_*k*_ (M)**	**Δf_*k*_ (N)**	**$P_{k}^{+}$ (M)**	**$P_{k}^{-}$ (M)**	**$P_{k}^{+}$ (H)**	**$P_{k}^{-}$ (H)**
1N	1.95	0.17	0.0265	0.0052	0.0355	0.0013
2C	12.26	9.75	0.2046	−0.0477	0.1282	−0.0128
3C	−2.86	−3.50	−0.0495	0.0454	0.0107	0.0382
4N	−4.84	−3.07	−0.0093	0.0664	−0.0078	0.0502
6N	−4.90	−4.93	−0.0386	0.1255	−0.0014	0.0842
7C	4.31	3.52	0.0990	−0.0287	0.0724	0.0118
8N	−7.69	−5.30	0.0121	0.1827	0.0144	0.1373
10C	3.75	3.52	0.0606	−0.0093	0.0446	0.0055
11C	−4.19	−3.69	−0.0307	0.0601	0.0164	0.0672
13C	3.64	3.40	0.1828	0.0879	0.1057	0.0693
15C	−3.10	−0.39	−0.0496	0.0160	−0.0010	0.0274
18C	−1.20	−0.94	−0.0285	−0.0154	−0.0028	0.0082
20C	−2.43	−1.71	0.0724	0.1095	0.0502	0.0801
23C	8.06	7.25	0.2653	0.0842	0.1563	0.0720
25C	−3.21	−1.99	0.1020	0.1482	0.0709	0.1094
26C	−1.89	−1.47	−0.0388	−0.0136	−0.0033	0.0150
31C	−4.63	−0.94	−0.0663	0.0355	0.0001	0.0422
32O	1.16	1.07	0.0184	−0.0118	0.0194	0.0009
79N	2.58	1.75	0.0473	−0.0153	0.0355	−0.0113
81C	1.28	0.19	0.0000	−0.0043	0.0127	0.0010

**Table 7:** The condensed dual descriptor calculated with Mulliken atomic charges Δf_*k*_ (M), and with NPA atomic charges Δf_*k*_ (N), the electrophilic and nucleophilic Parr functions with Mulliken atomic spin densities $P_{k}^{+}$ (M) and $P_{k}^{-}$ (M), and the electrophilic and nucleophilic Parr functions with Hirshfeld (or CM5) atomic spin densities $P_{k}^{+}$ (H) and $P_{k}^{-}$ (H) for the BISARG(p) melanoidin molecule. Hydrogens and atomic sites where the absolute value of the dual descriptor is lower than 1 are not shown.

**Atom**	**Δf_*k*_ (M)**	**Δf_*k*_ (N)**	**$P_{k}^{+}$ (M)**	**$P_{k}^{-}$ (M)**	**$P_{k}^{+}$ (H)**	**$P_{k}^{-}$ (H)**
1N	−0.28	−1.09	−0.0083	0.0179	0.0158	0.0112
2C	12.50	8.97	0.2317	−0.0230	0.1412	−0.0021
4C	3.40	2.52	0.0402	−0.0084	0.0341	−0.0032
5N	2.40	2.14	0.0260	−0.0076	0.0234	−0.0062
8N	−1.30	−0.84	−0.0074	0.0218	−0.0056	0.0161
12N	−6.95	−3.44	0.0425	0.1817	0.0308	0.1445
15C	−10.14	−7.30	−0.0588	0.1222	−0.0089	0.1049
16O	−3.36	−4.03	0.0136	0.0797	0.0125	0.0662
17C	13.43	8.74	0.3116	0.0363	0.1795	0.0297
19C	−1.32	1.08	−0.0883	0.0093	−0.0042	0.0134
20C	6.25	4.80	0.1683	0.0409	0.1008	0.0299
21O	1.44	1.41	0.0184	−0.0043	0.0200	0.0002
24C	3.85	2.95	0.1294	0.0546	0.0900	0.0401
27C	2.39	4.54	0.1996	0.0718	0.1140	0.0759
29C	−7.81	−5.59	0.0807	0.1977	0.0566	0.1467
30C	−2.76	−2.17	−0.0284	−0.0127	−0.0017	0.0222
33C	−3.67	−2.45	0.1007	0.1274	0.0610	0.0975
35C	−7.01	−2.75	−0.0547	0.0663	−0.0039	0.0638

From the results for the local reactivity descriptors in Table 6, it can be concluded that C2 and C23 will be the preferred sites for a nucleophilic attack and that these atoms will act as electrophilic species in a chemical reaction. In turn, it can be appreciated that N8 will be prone to electrophilic attacks and that this atomic site will act as a nucleophilic species in chemical reactions that involve the BISARG molecule in water. In turn, for the case of the BISARG(p) molecule in water, C2 and C17 will be the preferred sites for a nucleophilic attack while C15 and C29 will be the sites for electrophilic reactions.

## 5. Conclusions

Eight fixed RSH density functionals, including CAM-B3LYP, LC-ωPBE, M11, MN12SX, N12SX, ωB97, ωB97X, and ωB97XD, were examined to determine whether they fulfill the empirical KID procedure so as to provide computational support for this common practice. The assessment was conducted by comparing the values from HOMO and LUMO calculations to those generated by the ΔSCF technique for the BISARG molecule and its protonated derivative, BISARG(p). BISARG and BISARG(p) are intermediate melanoidin pigments that are of academic and industrial interest. The study has observed that the range-separated and hybrid meta-NGA density functionals tend to be the most suited in meeting this goal. Thus, they can be suitable alternatives to density functionals where the behavior of them is optimally tuned using a gap-fitting procedure. They also exhibit the desirable prospect of benefiting future studies aimed at understanding the chemical reactivity of colored melanoidins with larger molecular weights when reducing sugars react with proteins and peptides.

It is not the goal of Computational Chemistry to perform studies to reproduce known experimental results except in the case that they can be used for the calibration of a particular technique. Instead, it can be useful to predict in advance the structural and chemical reactivity characteristics of new or unknown molecular systems whose properties have not been reported and as guide for future research. As far as we know, there are no reports in the literature about the chemical reactivity properties for the molecular systems considered in this work and it is not possible to perform any kind of comparisons. However, the present study shows that with an adequate choice of the model chemistry we have been able to predict the sites of interaction of the BISARG and BISARG(p) molecules with impressive accuracy starting from the knowledge of the HOMO, LUMO, and HOMO-LUMO gap energies of the studied systems. This involves having DFT-based reactivity descriptors, including Fukui functions, Parr functions, and Dual Descriptor calculations. In conclusion, the Conceptual DFT descriptors are useful in characterizing and describing the preferred reactive sites and in comprehensively explaining the reactivity of the molecules.

## Author contributions

DG-M conceived and designed the research and headed, wrote and revised the manuscript, while JF contributed to the writing and the revision of the article.

## Acknowledgments

This work has been partially supported by CIMAV, SC, and Consejo Nacional de Ciencia y Tecnología (CONACYT, Mexico) through Grant 219566-2014 for Basic Science Research. DG-M conducted this work while a Visiting Lecturer at the University of the Balearic Islands from which support is gratefully acknowledged. This work was cofunded by the Ministerio de Economía y Competitividad (MINECO) and the European Fund for Regional Development (FEDER) (CTQ2014-55835-R).

### Conflict of interest statement

The authors declare that the research was conducted in the absence of any commercial or financial relationships that could be construed as a potential conflict of interest.
