# Supplementary material for: Conceptual DFT Study of the Local Chemical Reactivity of the Colored BISARG Melanoidin and Its Protonated Derivative
Source: Front Chem. 2018 May 1;6:136. doi: 10.3389/fchem.2018.00136 (PMC5938602; doi:10.3389/fchem.2018.00136)
Supplement: Supplementary file 1 [file Table_1.pdf]

Supplementary Material:
Conceptual DFT Study of the Local Chemical Reactivity of the Colored BISARG Melanoidin and its Protonated Derivative

Juan Frau and Daniel Glossman-Mitnik*
*Correspondence:
Daniel Glossman-Mitnik:
daniel.glossman@cimav.edu.mx

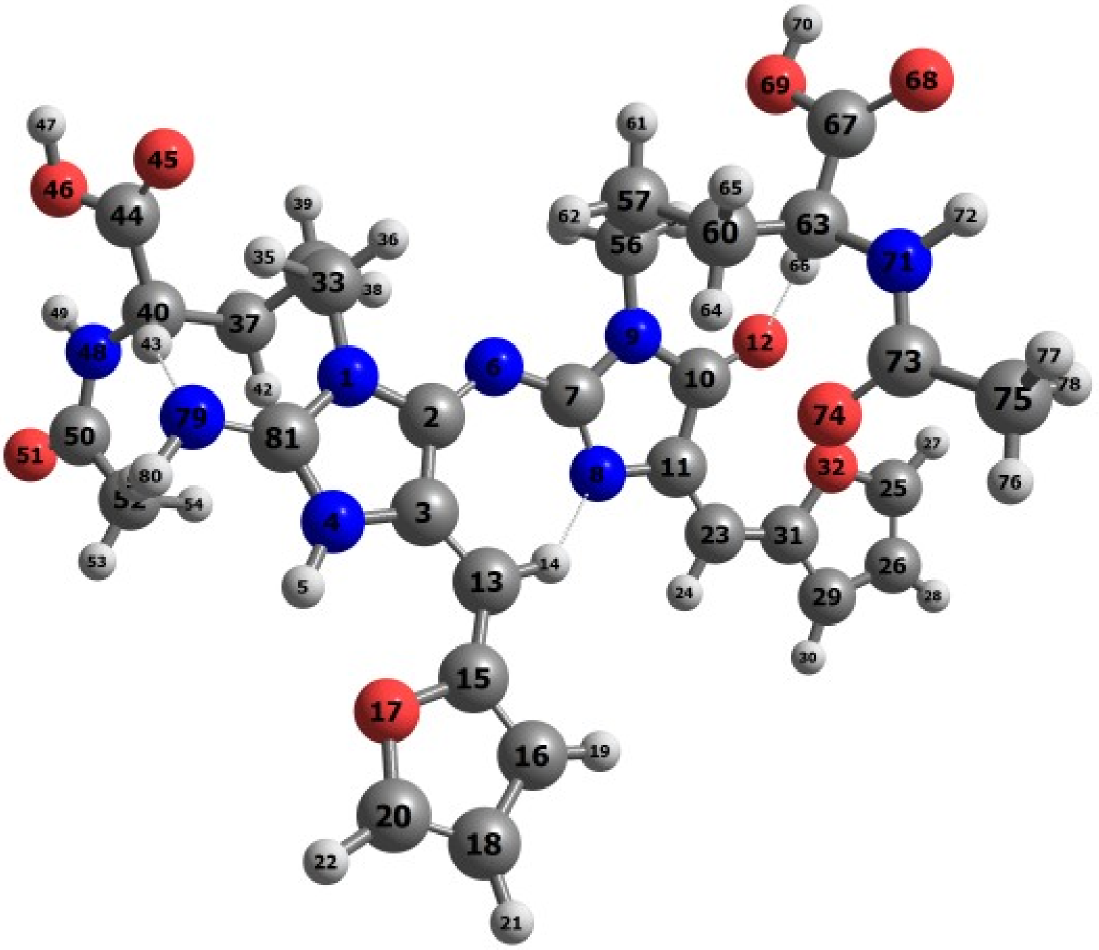

Supplementary Figure 1. An schematic representation of the optimized structure of the BISARG melanoidin molecule calculated with the MN12SX density functional and the Def2SVP basis set using water as solvent simulated with the SMD solvation model showing the numbering of the atoms.

Supplementary Table 1. Calculated bond lengths (in Å) of the BISARG intermediate melanoidin pigment with the MN12SX density functional and the Def2SVP basis set using water as solvent simulated with the SMD solvation model.

Bond	Distance	Bond	Distance	Bond	Distance	Bond	Distance
R(1-2)	1.383	R(16-18)	1.422	R(37-40)	1.549	R(60-63)	1.542
R(1-33)	1.446	R(16-19)	1.094	R(37-41)	1.109	R(60-64)	1.108
R(1-81)	1.387	R(17-20)	1.381	R(37-42)	1.109	R(60-65)	1.109
R(2-3)	1.479	R(18-20)	1.365	R(40-43)	1.139	R(63-66)	1.132
R(2-6)	1.321	R(18-21)	1.094	R(40-44)	1.518	R(63-67)	1.512
R(3-4)	1.396	R(20-22)	1.096	R(40-48)	1.446	R(63-71)	1.450
R(3-13)	1.366	R(23-24)	1.102	R(44-45)	1.223	R(67-68)	1.225
R(4-5)	1.031	R(23-31)	1.429	R(44-46)	1.383	R(67-69)	1.379
R(4-81)	1.382	R(25-26)	1.370	R(46-47)	0.982	R(69-70)	0.982
R(6-7)	1.369	R(25-27)	1.096	R(48-49)	1.034	R(71-72)	1.036
R(7-8)	1.335	R(25-32)	1.366	R(48-50)	1.379	R(71-73)	1.374
R(7-9)	1.388	R(26-28)	1.094	R(50-51)	1.236	R(73-74)	1.237
R(8-11)	1.394	R(26-29)	1.418	R(50-52)	1.515	R(73-75)	1.518
R(9-10)	1.393	R(29-30)	1.094	R(52-53)	1.097	R(75-76)	1.096
R(9-56)	1.441	R(29-31)	1.385	R(52-54)	1.102	R(75-77)	1.101
R(10-11)	1.491	R(31-32)	1.382	R(52-55)	1.102	R(75-78)	1.101
R(10-12)	1.241	R(33-34)	1.529	R(56-57)	1.530	R(79-80)	1.038
R(11-23)	1.369	R(33-35)	1.109	R(56-58)	1.111	R(79-81)	1.302
R(13-14)	1.110	R(33-36)	1.114	R(56-59)	1.116	R(8-14)	1.931
R(13-15)	1.434	R(34-37)	1.524	R(57-60)	1.524	R(12-66)	1.980
R(15-16)	1.380	R(34-38)	1.108	R(57-61)	1.108	R(43-79)	1.970
R(15-17)	1.396	R(34-39)	1.106	R(57-62)	1.109		

Supplementary Table 2. Calculated bond angles (in °) of the BISARG intermediate melanoidin pigment with the MN12SX density functional and the Def2SVP basis set using water as solvent simulated with the SMD solvation model.

Bond	Angle	Bond	Angle	Bond	Angle	Bond	Angle
A(2-1-33)	124.2	A(11-23-24)	117.2	A(34-37-41)	108.8	A(61-57-62)	106.8
A(2-1-81)	111.4	A(11-23-31)	127.6	A(34-37-42)	109.4	A(63-60-64)	106.6
A(1-2-3)	105.7	A(14-13-15)	117.9	A(38-34-39)	107.0	A(63-60-65)	109.3
A(1-2-6)	117.9	A(13-14-8)	140.7	A(40-37-41)	109.1	A(60-63-66)	108.5
A(33-1-81)	124.0	A(13-15-16)	130.4	A(40-37-42)	108.1	A(60-63-67)	113.7
A(1-33-34)	112.6	A(13-15-17)	120.7	A(37-40-43)	107.6	A(60-63-71)	109.7
A(1-33-35)	108.2	A(16-15-17)	108.9	A(37-40-44)	111.2	A(64-60-65)	107.0
A(1-33-36)	108.1	A(15-16-18)	107.1	A(37-40-48)	110.7	A(66-63-67)	108.5
A(1-81-4)	106.5	A(15-16-19)	126.1	A(41-37-42)	106.4	A(66-63-71)	109.8
A(1-81-79)	124.4	A(15-17-20)	106.7	A(43-40-44)	107.5	A(63-66-12)	167.2
A(3-2-6)	136.2	A(18-16-19)	126.7	A(43-40-48)	110.4	A(67-63-71)	106.6
A(2-3-4)	105.2	A(16-18-20)	107.0	A(40-43-79)	161.7	A(63-67-68)	124.1
A(2-3-13)	130.5	A(16-18-21)	126.6	A(44-40-48)	109.4	A(63-67-69)	115.9
A(2-6-7)	125.4	A(17-20-18)	110.2	A(40-44-45)	125.2	A(63-71-72)	115.7
A(4-3-13)	124.4	A(17-20-22)	117.4	A(40-44-46)	115.3	A(63-71-73)	122.0
A(3-4-5)	123.5	A(20-18-21)	126.3	A(40-48-49)	116.9	A(68-67-69)	120.0
A(3-4-81)	111.2	A(18-20-22)	132.5	A(40-48-50)	126.8	A(67-69-70)	107.4
A(3-13-14)	116.9	A(24-23-31)	115.2	A(45-44-46)	119.5	A(72-71-73)	120.9
A(3-13-15)	125.2	A(23-31-29)	129.8	A(44-46-47)	107.5	A(71-73-74)	121.9
A(5-4-81)	125.3	A(23-31-32)	121.2	A(49-48-50)	116.2	A(71-73-75)	115.1
A(4-81-79)	129.1	A(26-25-27)	132.1	A(48-50-51)	119.2	A(74-73-75)	122.9
A(6-7-8)	129.9	A(26-25-32)	110.3	A(48-50-52)	118.7	A(73-75-76)	110.4
A(6-7-9)	118.4	A(25-26-28)	126.6	A(51-50-52)	122.1	A(73-75-77)	110.7
A(8-7-9)	111.6	A(25-26-29)	106.6	A(50-52-53)	109.6	A(73-75-78)	110.5
A(7-8-11)	108.0	A(27-25-32)	117.6	A(50-52-54)	110.1	A(76-75-77)	108.8
A(7-8-14)	110.9	A(25-32-31)	107.3	A(50-52-55)	111.9	A(76-75-78)	108.6
A(7-9-10)	108.8	A(28-26-29)	126.8	A(53-52-54)	107.9	A(77-75-78)	107.7
A(7-9-56)	126.9	A(26-29-30)	126.8	A(53-52-55)	109.3	A(80-79-81)	112.7
A(8-11-10)	107.6	A(26-29-31)	106.8	A(54-52-55)	107.9	A(80-79-43)	124.3
A(8-11-23)	121.2	A(30-29-31)	126.4	A(57-56-58)	110.5	A(81-79-43)	109.4
A(11-8-14)	129.8	A(29-31-32)	109.0	A(57-56-59)	109.2		
A(10-9-56)	124.3	A(34-33-35)	110.5	A(56-57-60)	113.8		
A(9-10-11)	104.2	A(34-33-36)	108.8	A(56-57-61)	108.4		
A(9-10-12)	122.6	A(33-34-37)	114.3	A(56-57-62)	108.9		
A(9-56-57)	112.5	A(33-34-38)	109.1	A(58-56-59)	107.8		
A(9-56-58)	107.1	A(33-34-39)	107.8	A(60-57-61)	110.4		
A(9-56-59)	109.6	A(35-33-36)	108.5	A(60-57-62)	108.3		
A(11-10-12)	133.3	A(37-34-38)	108.6	A(57-60-63)	115.2		
A(10-11-23)	131.2	A(37-34-39)	109.7	A(57-60-64)	109.4		
A(10-12-66)	99.0	A(34-37-40)	114.8	A(57-60-65)	109.0		

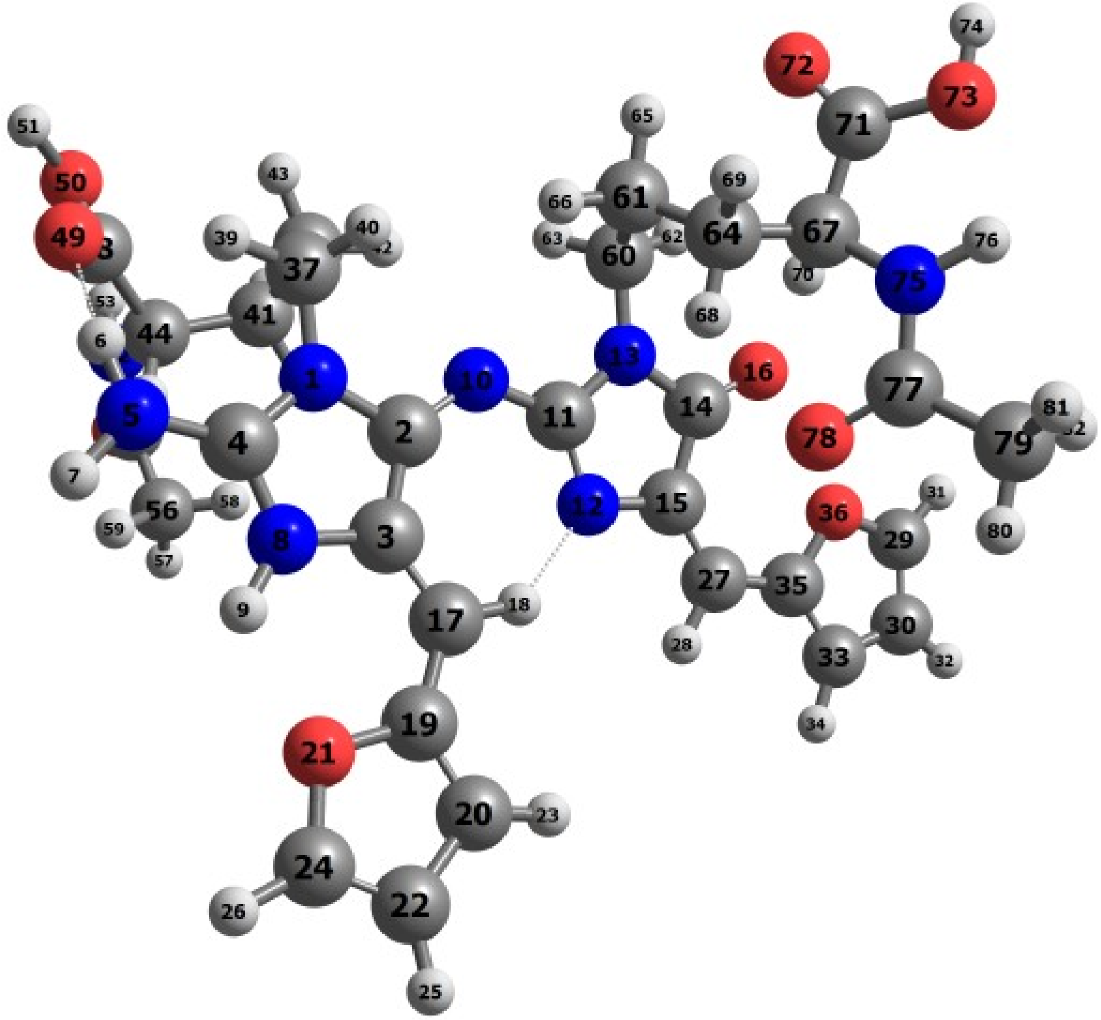

Supplementary Figure 2. An schematic representation of the optimized structure of the protonated BISARG(p) melanoidin molecule calculated with the MN12SX density functional and the Def2SVP basis set using water as solvent simulated with the SMD solvation model showing the numbering of the atoms.

Supplementary Table 3. Calculated bond lengths (in Å) of the protonated BISARG(p) intermediate melanoidin pigment with the MN12SX density functional and the Def2SVP basis set using water as solvent simulated with the SMD solvation model.

Bond	Distance	Bond	Distance	Bond	Distance	Bond	Distance
R(1-2)	1.416	R(17-19)	1.423	R(38-41)	1.526	R(61-64)	1.523
R(1-4)	1.355	R(19-20)	1.384	R(38-42)	1.107	R(61-65)	1.107
R(1-37)	1.450	R(19-21)	1.403	R(38-43)	1.107	R(61-66)	1.111
R(2-3)	1.460	R(20-22)	1.417	R(41-44)	1.552	R(64-67)	1.541
R(2-10)	1.318	R(20-23)	1.094	R(41-45)	1.106	R(64-68)	1.108
R(3-8)	1.412	R(21-24)	1.379	R(41-46)	1.108	R(64-69)	1.109
R(3-17)	1.374	R(22-24)	1.368	R(44-47)	1.121	R(67-70)	1.130
R(4-5)	1.351	R(22-25)	1.093	R(44-48)	1.521	R(67-71)	1.515
R(4-8)	1.351	R(24-26)	1.096	R(44-52)	1.446	R(67-75)	1.452
R(5-6)	1.055	R(27-28)	1.102	R(48-49)	1.239	R(71-72)	1.221
R(5-7)	1.028	R(27-35)	1.419	R(48-50)	1.349	R(71-73)	1.385
R(8-9)	1.034	R(29-30)	1.376	R(50-51)	0.983	R(73-74)	0.983
R(10-11)	1.367	R(29-31)	1.096	R(52-53)	1.036	R(75-76)	1.033
R(11-12)	1.341	R(29-36)	1.359	R(52-54)	1.397	R(75-77)	1.377
R(11-13)	1.382	R(30-32)	1.093	R(54-55)	1.223	R(77-78)	1.239
R(12-15)	1.385	R(30-33)	1.410	R(54-56)	1.517	R(77-79)	1.515
R(13-14)	1.398	R(33-34)	1.094	R(56-57)	1.096	R(79-80)	1.096
R(13-60)	1.444	R(33-35)	1.392	R(56-58)	1.101	R(79-81)	1.100
R(14-15)	1.492	R(35-36)	1.385	R(56-59)	1.101	R(79-82)	1.100
R(14-16)	1.235	R(37-38)	1.532	R(60-61)	1.531	R(6-49)	1.746
R(15-27)	1.377	R(37-39)	1.107	R(60-62)	1.110		
R(17-18)	1.114	R(37-40)	1.112	R(60-63)	1.116		

Supplementary Table 4. Calculated bond angles (in °) of the BISARG intermediate melanoidin pigment with the MN12SX density functional and the Def2SVP basis set using water as solvent simulated with the SMD solvation model.

Bond	Angle	Bond	Angle	Bond	Angle	Bond	Angle
R(12-18)	1.917	A(13-60-62)	106.9	A(39-37-40)	107.7	A(64-61-66)	108.5
A(2-1-4)	110.2	A(13-60-63)	109.6	A(41-38-42)	107.4	A(61-64-67)	114.0
A(2-1-37)	123.3	A(15-14-16)	133.2	A(41-38-43)	109.7	A(61-64-68)	109.5
A(1-2-3)	105.2	A(14-15-27)	131.2	A(38-41-44)	116.6	A(61-64-69)	109.1
A(1-2-10)	116.7	A(15-27-28)	117.2	A(38-41-45)	107.6	A(65-61-66)	107.3
A(4-1-37)	126.1	A(15-27-35)	127.4	A(38-41-46)	109.4	A(67-64-68)	107.3
A(1-4-5)	125.9	A(18-17-19)	118.8	A(42-38-43)	106.7	A(67-64-69)	109.5
A(1-4-8)	108.9	A(17-18-12)	141.1	A(44-41-45)	108.3	A(64-67-70)	108.3
A(1-37-38)	113.2	A(17-19-20)	130.6	A(44-41-46)	108.2	A(64-67-71)	111.8
A(1-37-39)	108.3	A(17-19-21)	120.8	A(41-44-47)	108.6	A(64-67-75)	109.7
A(1-37-40)	108.0	A(20-19-21)	108.7	A(41-44-48)	112.3	A(68-64-69)	107.2
A(3-2-10)	138.1	A(19-20-22)	107.3	A(41-44-52)	111.2	A(70-67-71)	107.7
A(2-3-8)	105.2	A(19-20-23)	125.9	A(45-41-46)	106.3	A(70-67-75)	108.8
A(2-3-17)	131.5	A(19-21-24)	106.6	A(47-44-48)	107.0	A(71-67-75)	110.4
A(2-10-11)	125.6	A(22-20-23)	126.8	A(47-44-52)	109.0	A(67-71-72)	125.1
A(8-3-17)	123.3	A(20-22-24)	107.1	A(48-44-52)	108.7	A(67-71-73)	115.0
A(3-8-4)	110.6	A(20-22-25)	126.6	A(44-48-49)	123.2	A(67-75-76)	117.0
A(3-8-9)	122.8	A(21-24-22)	110.3	A(44-48-50)	116.0	A(67-75-77)	120.2
A(3-17-18)	115.9	A(21-24-26)	117.3	A(44-52-53)	115.4	A(72-71-73)	119.9
A(3-17-19)	125.3	A(24-22-25)	126.3	A(44-52-54)	123.6	A(71-73-74)	107.6
A(5-4-8)	125.2	A(22-24-26)	132.3	A(49-48-50)	120.9	A(76-75-77)	119.5
A(4-5-6)	119.9	A(28-27-35)	115.4	A(48-49-6)	119.3	A(75-77-78)	121.6
A(4-5-7)	118.8	A(27-35-33)	130.0	A(48-50-51)	109.3	A(75-77-79)	115.4
A(4-8-9)	126.6	A(27-35-36)	121.3	A(53-52-54)	114.8	A(78-77-79)	123.0
A(6-5-7)	118.2	A(30-29-31)	131.9	A(52-54-55)	119.3	A(77-79-80)	110.5
A(5-6-49)	166.2	A(30-29-36)	110.5	A(52-54-56)	117.6	A(77-79-81)	110.7
A(10-11-12)	129.4	A(29-30-32)	126.5	A(55-54-56)	122.9	A(77-79-82)	110.5
A(10-11-13)	118.9	A(29-30-33)	106.6	A(54-56-57)	109.6	A(80-79-81)	108.8
A(12-11-13)	111.6	A(31-29-36)	117.6	A(54-56-58)	111.2	A(80-79-82)	108.6
A(11-12-15)	107.9	A(29-36-35)	107.3	A(54-56-59)	110.9	A(81-79-82)	107.7
A(11-12-18)	116.1	A(32-30-33)	126.8	A(57-56-58)	108.4		
A(11-13-14)	108.7	A(30-33-34)	126.9	A(57-56-59)	108.6		
A(11-13-60)	127.1	A(30-33-35)	106.9	A(58-56-59)	108.0		
A(12-15-14)	107.8	A(34-33-35)	126.2	A(61-60-62)	110.6		
A(12-15-27)	121.0	A(33-35-36)	108.7	A(61-60-63)	109.5		
A(15-12-18)	133.3	A(38-37-39)	110.9	A(60-61-64)	113.3		
A(14-13-60)	124.2	A(38-37-40)	108.6	A(60-61-65)	108.5		
A(13-14-15)	104.0	A(37-38-41)	116.5	A(60-61-66)	109.1		
A(13-14-16)	122.8	A(37-38-42)	108.5	A(62-60-63)	107.7		
A(13-60-61)	112.5	A(37-38-43)	107.6	A(64-61-65)	110.0		
